# DNA methylation analysis of floral parts revealed dynamic changes during the development of homostylous *Fagopyrum tataricum* and heterostylous *F. esculentum* flowers

**DOI:** 10.1186/s12870-024-05162-w

**Published:** 2024-05-23

**Authors:** Katarzyna Sala-Cholewa, Alicja Tomasiak, Katarzyna Nowak, Artur Piński, Alexander Betekhtin

**Affiliations:** Institute of Biology, Biotechnology and Environmental Protection, Faculty of Natural Sciences, University of Silesia in Katowice, 28 Jagiellonska St, Katowice, 40-032 Poland

**Keywords:** DNA methylation, Epigenetics, *Fagopyrum esculentum*, *Fagopyrum tataricum*, Flowers, Gene expression, Heterostyly

## Abstract

**Background:**

Proper flower development is essential for plant reproduction, a crucial aspect of the plant life cycle. This process involves precisely coordinating transcription factors, enzymes, and epigenetic modifications. DNA methylation, a ubiquitous and heritable epigenetic mechanism, is pivotal in regulating gene expression and shaping chromatin structure. *Fagopyrum esculentum* demonstrates anti-hypertensive, anti-diabetic, anti-inflammatory, cardio-protective, hepato-protective, and neuroprotective properties. However, the heteromorphic heterostyly observed in *F. esculentum* poses a significant challenge in breeding efforts. *F. tataricum* has better resistance to high altitudes and harsh weather conditions such as drought, frost, UV-B radiation damage, and pests. Moreover, *F. tataricum* contains significantly higher levels of rutin and other phenolics, more flavonoids, and a balanced amino acid profile compared to common buckwheat, being recognised as functional food, rendering it an excellent candidate for functional food applications.

**Results:**

This study aimed to compare the DNA methylation profiles between the Pin and Thrum flower components of *F. esculentum*, with those of self-fertile species of *F. tataricum*, to understand the potential role of this epigenetic mechanism in *Fagopyrum* floral development. Notably, *F. tataricum* flowers are smaller than those of *F. esculentum* (Pin and Thrum morphs). The decline in DNA methylation levels in the developed open flower components, such as petals, stigmas and ovules, was consistent across both species, except for the ovule in the Thrum morph. Conversely, Pin and Tartary ovules exhibited a minor decrease in DNA methylation levels. The highest DNA methylation level was observed in Pin stigma from closed flowers, and the most significant decrease was in Pin stigma from open flowers. In opposition, the nectaries of open flowers exhibited higher levels of DNA methylation than those of closed flowers. The decrease in DNA methylation might correspond with the downregulation of genes encoding methyltransferases.

**Conclusions:**

Reduced overall DNA methylation and the expression of genes associated with these epigenetic markers in fully opened flowers of both species may indicate that demethylation is necessary to activate the expression of genes involved in floral development.

**Supplementary Information:**

The online version contains supplementary material available at 10.1186/s12870-024-05162-w.

## Background

Buckwheat constitutes a small genus of 22 members [1]. Two of the most widely cultivated species of this genus are *Fagopyrum esculentum* Moench (common buckwheat) and *Fagopyrum tataricum* (Tartary buckwheat) [2, 3]. Common buckwheat is commonly cultivated in Russia, China, France, Poland, and the American regions [4, 5]. In terms of overall yield, buckwheat is a minor crop. However, its popularity among consumers steadily increases due to its remarkable properties. Common buckwheat is rich in beneficial phenolic compounds like rutin, quercetin and C-glycosyl flavones (orientin, isoorientin and vitexin). Additionally, it is gluten-free and provides amino acids, dietary fibre, resistant starch, and vitamins [6–8]. It was demonstrated that *F. esculentum* exhibits anti-hypertension, anti-diabetic, anti-inflammatory, cardio-protective, hepato-protective, and neuroprotective properties [7, 8]. Tartary buckwheat exhibits higher than common buckwheat resistance to high altitudes, harsh weather conditions such as drought, frost, UV-B radiation damage, and pests [9]. Additionally, compared to common buckwheat, *F. tataricum* contains more rutin and other phenolics and flavonoids, with well-balanced amino acids, making it a functional pseudocereal [10]. One significant advantage of Tartary buckwheat lies in the differences in flower structure and pollination methods, which directly impact yield. *F. esculentum* is an obligatory cross-pollinating, heterostylous species, while *F. tataricum* is self-pollinating, homostylous species eliminating reliance on external pollinators [11, 12]. *F. esculentum* has two floral morphs: Pin and Thrum, which differ in stamen and style length ratio, amount and size of the produced pollen grain, exine sculpturing, and nectar production [13, 14]. The distyly of *F. esculentum* is controlled by a cluster of genes, i.e. supergene S [13, 15]. Recently, the *S-LOCUS EARLY FLOWERING 3* gene (*S-ELF3*), present within the S supergene locus, was shown to control style length and style incompatibility. The inactivation of *S-ELF3* has led to the breakage of heterostyly and successful pollination [15]. While in the incompatible intra-morph pollination, the pollen tube growth within the style is inhibited [13, 16, 17].

Proper flower development ensures reproduction, one of the most vital parts of the plant’s life cycle. This process relies on fine-tuned machinery of transcription factors, enzymes and epigenetic modifications to function correctly. DNA methylation, a universal and heritable epigenetic mechanism, regulates gene expression and the chromatin structure [16, 18]. The extent of DNA methylation significantly shapes the plant genome’s structure and function. Various molecular mechanisms are impacted by DNA methylation, highlighting its pivotal role in regulating cellular processes within plants [16, 19–22]. DNA methylation takes place at CG, CHG and CHH sites (H = A, T or C); methylation at these three sites is achieved by *DNA METHYLTRANSFERASE 1* (*MET1*), the plant-specific *CHROMOMETHYLASE 3* (*CMT3*), and *DOMAINS REARRANGED METHYLTRANSFERASEs* (*DRMs*), respectively [23, 24]. Each type is crucial for development and the ability to respond to environmental stresses [16, 25]. An opposite phenomenon, demethylation, is dependent on four bifunctional 5-methylcytosine glycosylases: *REPRESSOR OF SILENCING 1* (*ROS1*), *DEMETER* (*DME*), *DME-LIKE 2* (*DML2*), and *DML3*, which are involved in the removal of methylated bases and cleavage of the DNA backbone at abasic sites [26]. *ROS1* counteracts the DNA methylation pathway to prevent plant gene silencing [27]. Research demonstrated that DNA methylation plays an essential role in flower development, with demethylation dominating during early flower development [28]. It was also reported that decreased levels of DNA methylation induced early flowering and that demethylation activities are correlated with changes in the expression levels of DNA methylation genes [28–30]. On the other hand, experiments on *Azalea japonica* and *Arabidopsis* demonstrated an increase in global DNA methylation during the transition from the vegetative to the flowering stage [31, 32]. DNA methylation was determined to influence various aspects of flower development and morphology [33]. It affects floret closing in barley, and the development of bisexual flowers in *Populus cathayana* and *Fraxinus mandshurica* [34–36]. DNA methylation is also involved in forming double flowers and dichromatic petals [37, 38]. Moreover, it plays a crucial role in the expression of genes related to anthocyanin pigmentation in flower tissue [9, 39, 40]. Existing evidence suggests that methylation alterations accompany the flowering process in plants. Methylation regulates key flowering-related genes such as *SOC1, AP1*, and *SPL* and specific transcription factor genes, including *WUS* homeobox-containing (*WOX*) genes in apple [41].

While numerous processes related to DNA methylation in plant vegetative tissues have been extensively studied, there remains a scarcity of information regarding DNA methylation during flower development, particularly in flowers exhibiting distinct morphologies. Thus, this study aimed to compare the DNA methylation status of Pin and Thrum flower components of *F. esculentum* that participate in pollination and embryo production with self-sufficient species of *F. tataricum*.

## Methods

### Plant material

*F. tataricum* seeds, sample k-17, were obtained from the N. I. Vavilov Institute of Plant Genetic Resources, Saint Petersburg, Russia. K-17 sample is a widely cultivated landrace of *F. tataricum*. Seeds are available upon request from the authors. *F. esculentum* seeds of the Panda cultivar are commercially available and were supplied from the Malopolska Plant Breeding, Poland. Both plant species were grown in pots with soil mixed with vermiculite (3:1, w/v) in a greenhouse at 20 ± 1 °C, under a 16/8 h light/dark photoperiod, provided by lamps emitting white light at the intensity of 90 µmol m ^− 2^ s ^− 1^. After approximately three to four weeks, the flowers started to appear and were gradually collected, photographed with the use of Keyence VHX-970 F digital microscope (Japan) equipped with an ultra-small high-performance zoom lens VH-Z20R/Z20T and wide-area illumination adapter OP-87,298 and designated for further procedures.

For analysis, the closed flowers from both species were fixed in the intact state. Open flowers were dissected into parts that involved excised petals, stigmas and remains that contained ovaries and nectaries.

### Histological and immunostaining procedures

Closed and open flower components from *F. tataricum*, as well as closed and open Pin and Thrum flower components from *F. esculentum* were fixed in 4% paraformaldehyde (Sigma-Aldrich, USA) in 1x phosphate-buffered saline (PBS), pH 7.3 and placed in the vacuum desiccator for three hours (with 30 min intervals), after which the incubation at 4 °C overnight has followed. After approximately 24 h, the fixative was replaced with 1 × PBS (twice for 15 min) and followed by dehydration in a graded ethanol series diluted in 1xPBS solution in each concentration (10%, 30%, 50%, 70% and 90%) and 99,8% twice for 30 min each. Subsequently, the embedding procedure was performed according to Wolny et al., 2014 [42]. 5 μm thick sections were prepared using a HYRAX M40 rotary microtome (Zeiss, Oberkochen, Germany) and placed on polysine-coated microscope slides (Epredia, Netherlands). Next, the de-embedding procedure comprised of placing the slides in 99,8% ethanol three times for 10 min, followed by the rehydration in ethanol/ 1xPBS solutions: 90%, 50%v/v and, finally in 1xPBS for 10 min each. Such prepared slides were used for both, the immunostaining and histological analysis. For the histological analysis, slides were stained with 0.05% aqueous solution of Toluidine blue O (TBO, Sigma-Aldrich, USA) for approximately 10 min, rinsed and mounted with 50% glycerol/ distilled water solution (v/v). Observations and photographs of histological sections were performed with an Olympus BX43F microscope equipped with an Olympus XC50 digital camera.

The immunostaining method was previously established by Braszewska-Zalewska et al., 2013 [43]. After the wax de-embedding described above, samples underwent the 2 N HCl (Sigma-Aldrich, USA) digestion for 45 min to denature DNA. Following, the slides were incubated with 5% bovine serum albumin (BSA, Sigma-Aldrich, USA) in 1xPBS for 1 h in the humid chamber at room temperature. Next, the primary antibody diluted in 1% BSA in 1xPBS (1:100) was applied, and slides were incubated at 4^o^ C overnight (Table 1). After the incubation, the slides were washed three times in 1xPBS. Subsequently, a secondary antibody diluted in 1% BSA in 1xPBS (1:100) was applied, and samples were incubated at 37 ^o^ C in the humid chamber in the dark for 1 h. After the incubation, the slides were again washed three times in 1xPBS and nuclei were counterstained with 4’,6-diamidyno-2-fenyloindol (DAPI, 2.5 g/ml in Vectashield).

Graphical depiction of the experimental design is available in Additional Files, File 1.

**Table 1:** List of antibodies used in the immunostaining

Antibody	Catalogue number	Company
Anti-5-methylcytosine	ab73938	Abcam, UK
Goat anti-mouse IgG	ab150113	Abcam, UK

### Fluorescence intensity measurements and statistical analysis

Images were captured with the Olympus FV1000 confocal system (Olympus, Poland) equipped with an Olympus IX81 inverted microscope. Fluorescence of Alexa488 (excitation 488 nm, emission 500–600 nm) was acquired from a 60x Plan Apo oil-immersion objective lens (NA 1.35), a 50 mW 405 nm diode laser and a 100 mW multi-line argon ion laser (Melles Griot BV, the Netherlands). The confocal laser scanning microscope offers a significant advantage in obtaining high-resolution images of optical sections through fluorescently labelled nuclei (z-stacks) [44, 45]. The pixel-by-pixel image generation produces exceptional quality images [45], enabling visualization of fluorescence distribution corresponding to DNA methylation within the three-dimensional chromatin architecture of a nucleus. The nucleus area was used to standardise the fluorescence intensity, preventing any artificial positive correlation between intensity and nucleus size. Consequently, the fluorescence intensity-to-nucleus area ratio indicated the level of DNA methylation [46]. An axial series of two-dimensional fluorescence images of the optical sections through the nuclei (z-stacks) was collected with the use of two separate photomultipliers (R6357, Hamamatsu Photonics, Hamamatsu, Japan) set to work in the integration mode at 4 µs pixel dwell time and 12-bit signal digitisation (4096 intensity levels). The fluorescence intensity levels of Alexa488 fluorochrome were subsequently measured in the ImageJ version 1.53s software (Wayne Rasband, National Institutes of Health, USA). ImageJ is a Java-based image processing program developed at the National Institutes of Health and the Laboratory for Optical and Computational Instrumentation (LOCI, University of Wisconsin) [47–50]. It performs various complex tasks such as editing, processing, and analysing 8-bit colour and grayscale images. ImageJ calculates area and pixel value statistics of selections and intensity-thresholded objects defined by the user and offers standard image processing [51–53]. Images were converted to eight bits and segmented with the threshold value parameter. Alexa488 fluorescence intensity was calculated as the mean values from the Integrated Density parameter per one nucleus, which depicted the sum of all pixels within the region of interest. Results are presented in relative units. Raw data obtained from the images were further analysed using statistical analysis software. This study utilised R Studio, an integrated development environment for R, a statistical programming language [54]. R is widely recognized as a statistical language, dominating other programming languages in developing statistical tools. It offers various packages suitable for scientific data analysis, including agricolae (Statistical Procedures for Agricultural Research), which can be used to determine significant differences between means [55], as well as other packages dedicated to data visualisation [56]. R facilitates the reproducible process of summarizing data after statistical analysis and visualizing it in graph form [57]. 500 nuclei were analysed from petals; 1000 nuclei from the nectary; 1500 nuclei from the ovary, and 1000 nuclei from the stigma in open and closed *F. tataricum* flowers and *F. esculentum* Pin open and closed and Thrum open and closed flower type. Numerical data is included in Additional File 2. Fluorescence data analysis and plotting was performed in R, a software environment for statistical computing and graphics in R Studio 2022.12.0 Build 353 (Script included in Additional File 3), an integrated development environment for R [58, 59]. One-way ANOVA test with the R Stats package [59] was followed by Tukey’s HSD test at the significance level *p* ≤ 0.05. Standard errors were likewise calculated with the stats package. The package agricolae (Statistical Procedures for Agricultural Research) calculated significant differences between means [55]. Subsequently, data was plotted with packages dedicated to visualising data: ggplot2 [60] and ggpubr [56]. Letters on the graphs indicate statistically significant differences between samples. Full immunostaining procedure and the fluorescence intensity analysis is described for *Fagopyrum* species [61, 62].

### RNA isolation and real-time qPCR

Total RNA was isolated from the closed and open flowers and Thrum and Pin types of *F. esculentum* and *F. tataricum*. Total RNA was isolated using a FastPure Plant Total RNA Isolation Kit (Polysaccharides and polyphenolics-rich) (Vazyme Biotech, Red Maple Hi-tech Industry Park, Nanjing, PRC). RNA concentrations were measured using a Nano-Drop ND-1000 (NanoDrop Technologies, Wilmington, DE, USA). The DNA was removed from the RNA samples by digesting them with an RNase-free DNase Set (Qiagen, Hilden, Germany). The oligo-dT primers and a Maxima H Minus First Strand cDNA Synthesis Kit (Thermo Fisher Scientific, Waltham, MA, USA) were used to produce the cDNA. The obtained cDNA was diluted four-fold with water and used at a volume of 2 µl in a qPCR reaction. Analyses were performed in a 10 µl volume using a LightCycler® 480 SYBR Green I Master (Roche, Basel, Switzerland). The primers were designed based on *Fagopyrum esculentum* “Pintian4” and *Fagopyrum tataricum* “Pinku1” references genomes with Primer3Plus (Additional File 4) [63]. The control genes (*SAND, ACTIN*) had a constant expression level in all tissue samples. Analyses were performed using a LightCycler 480 (Roche, Basel, Switzerland) under the following reaction conditions: initial denaturation of 5 min at 95 °C, followed by 10 s at 95 °C, 20 s at a temperature specific for the primers, 10 s at 72 °C, repeated in 40 cycles. Denaturation for the melt curve analysis was conducted for 5 s at 95 °C, followed by 1 min at 65 °C and heating to 98 °C (0.1 °C/s for the fluorescence measurement). The Ct values were calculated using LinRegPCR software (version 11, Academic Medical Centre, Amsterdam, The Netherlands). The plant tissues for the Real-Time qPCR analysis were produced in three biological repetitions, and two technical replicates of each repetition were analysed. The relative expression level was calculated using 2^–∆∆CT^, where ∆∆C_T_ represents ∆C_T_^reference condition^ − ∆C_T_^compared condition^.

### Statistical analysis

The Student t-test and one-way ANOVA (*p* < 0.05) followed by Tukey’s honestly-significant-difference test (Tukey HSD-test) (*p* < 0.05) were used to calculate any significant differences between the experimental combinations. The graphs show the average values with the standard error (SE) in Fig. 4 and the standard deviation (SD) in Fig. 5.

### Graphics

Photographs were cropped, adjusted (brightness, contrast) and arranged into figures in the Corel Draw 2020 program. A graphical depiction of the experimental design (Additional File 1) was prepared with BioRender (available online at: https://app.biorender.com/*).* Publication licences are included as Additional File 5.

## Results

### Anatomy and histology of the flowers

**Fig. 1:**
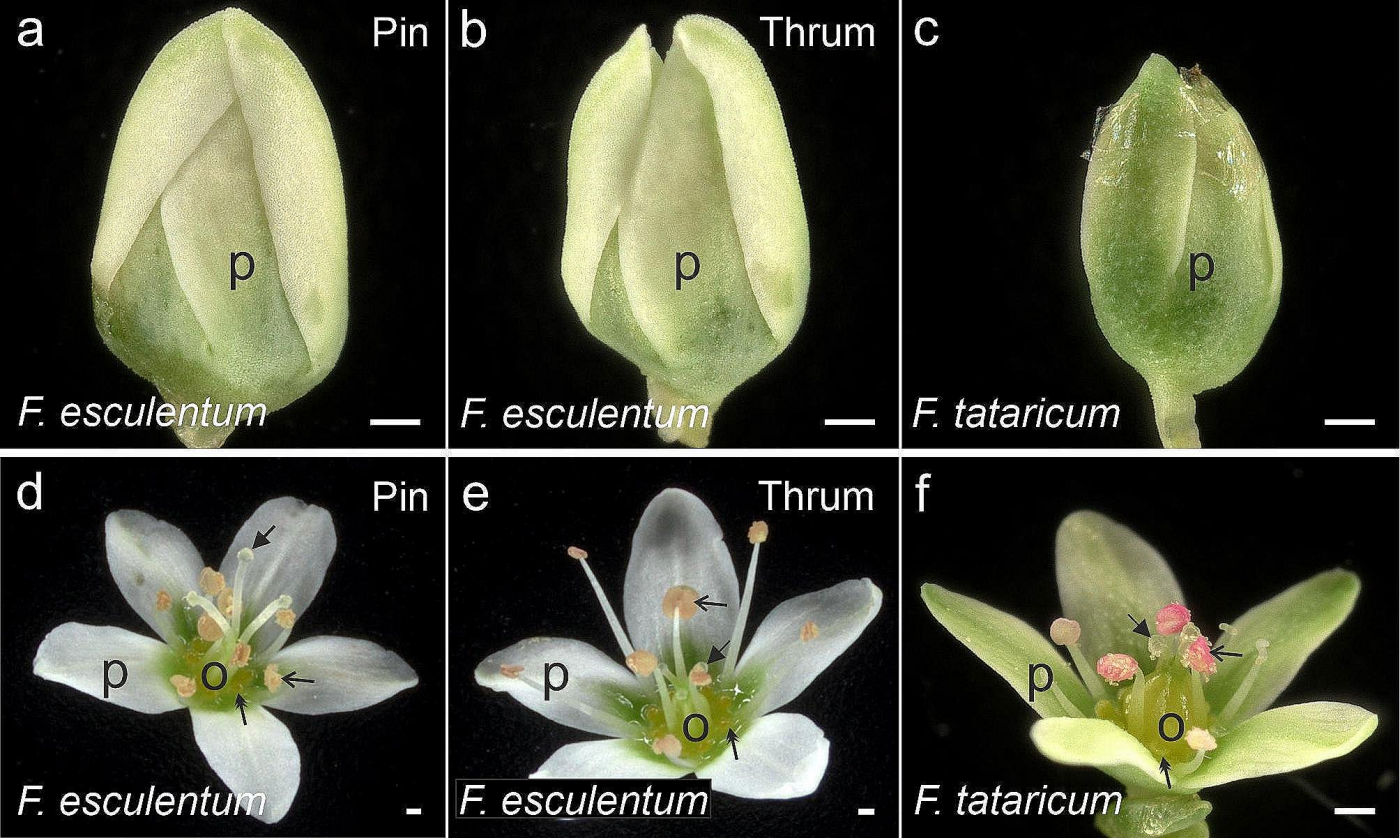
Morphology of analysed flowers. *F. esculentum* (a, d) Pin, (b, e) Thrum, and (c, f) *F*. tataricum. (**a** – **c**) closed flowers were tightly coiled in petals. (**d** – **f**) In open flowers, the anatomy is easily visible. Arrows – stigmas, double arrows – nectaries, open arrows – anthers, o – ovaries, p – petals. Scale bars: (a–f) = 300 μm

*F. tataricum* flowers, closed and open, were smaller than those of *F. esculentum* (Pin, Thrum) (Fig. 1). Closed flowers were coiled in petals of a greenish colour (Fig. 1a–c). In open flowers, the differences between two morphs were disclosed: Pin flowers featured a long style and shorter filaments (Fig. 1d), while Thrum had a short style and longer filaments (Fig. 1e). Open flowers of *F. tataricum* had green petals, with style and filaments of similar length (Fig. 1f).

In anthers of closed flowers, the developmental stage of pollen was advanced, with microspores enwrapped in egzine sheath, tapetum cells were already undergoing programmed cell death (Additional File 6a-c). The anthers of open flowers were fully developed and open, with mature pollen and no tapetum cell remains (Additional File 6d-f). Thus, the anthers were not considered in DNA methylation analysis due to the lack of reference (closed vs. open).

**Fig. 2:**
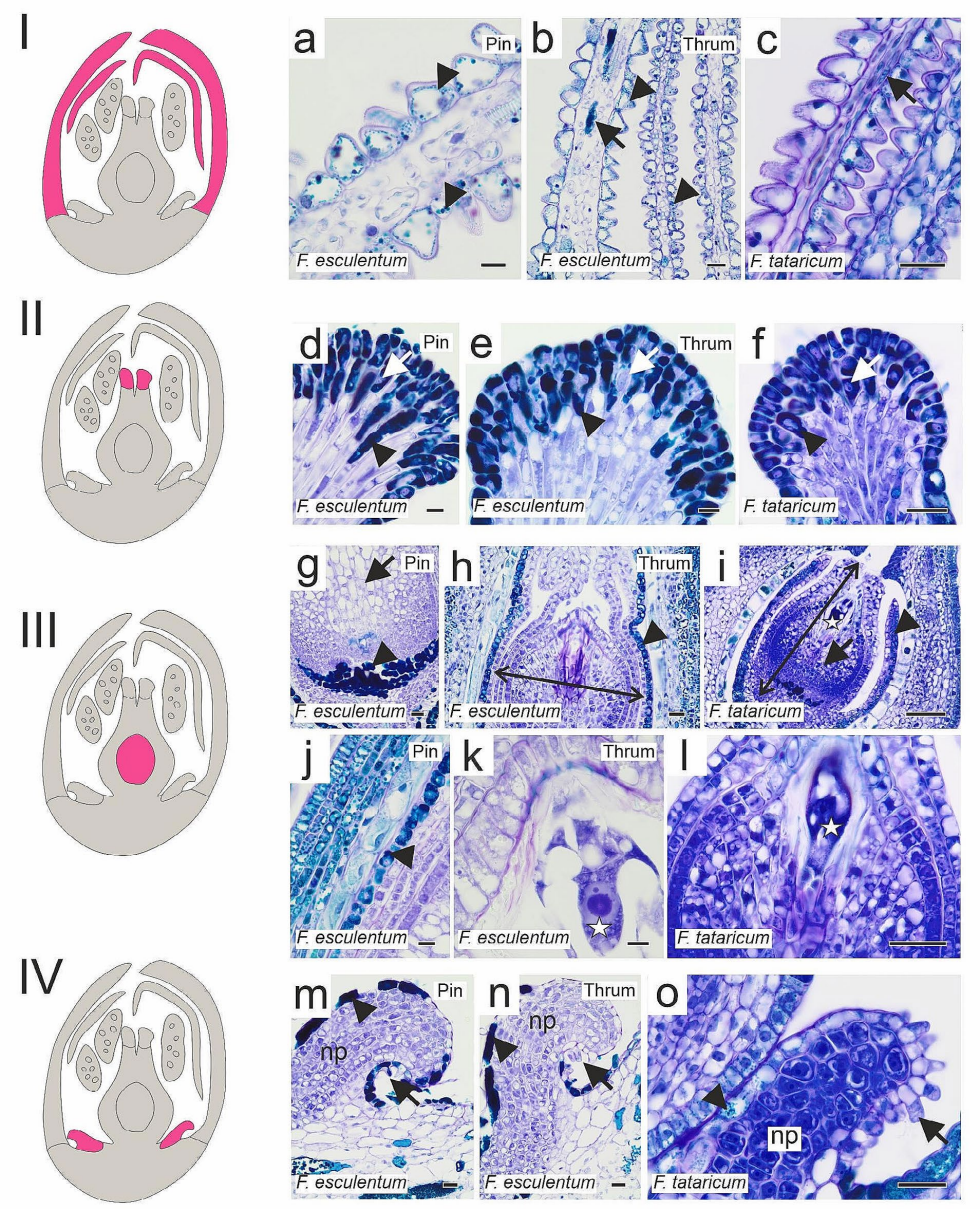
Histology of closed flower components (*F. esculentum* Pin, Thrum; *F. tataricum*). Schematic diagrams represent analysed flower components: I petals, II stigma, III ovary with ovule, IV nectary, marked in pink on the diagram. (**a** - **c**) petals, abundant polyphenols occurrence in epidermis (arrowheads) and mesophyll cells (arrows). (**d** - **f**) stigmas, papillae and subpapillae cells rich in polyphenols (arrowheads) but with visible nuclei (arrows). (**g** - **l**) ovaries and ovules, polyphenols present in basal part of ovule and in outer integument cells (arrowheads), arrows – nucellus parenchyma cells, embryo sacs (asterisks); double arrow stands for the extent of cells that were taken into account in the measurement of Alexa488 fluorescence intensity. (**m** - **o**) nectaries, epidermis rich in polyphenols (arrowheads), secretory trichomes (arrows), np nectary parenchyma. Scale bars: a, d, e, j, k = 10 μm; b, f, g, h, l, m – o = 20 μm; c, i = 50 μm

**Fig. 3:**
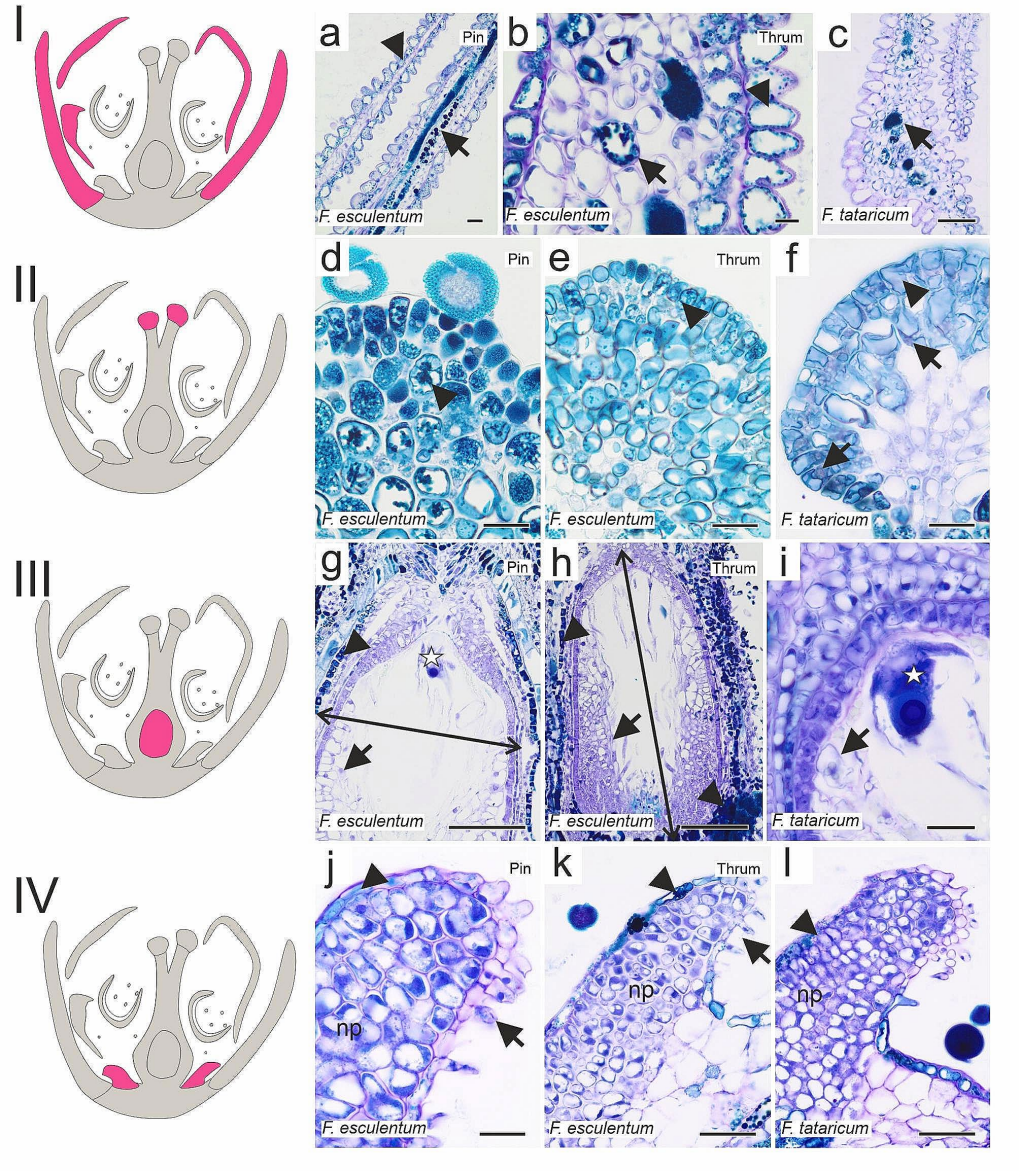
Histology of open flower components (*F. esculentum* Pin, Thrum; *F. tataricum*). Schematic diagrams represent analysed flower components: I petals, II stigma, III ovary with ovule, IV nectary, marked in pink on the diagram. (**a** – **c**) petals, abundant occurrence of polyphenols in the epidermis (arrowheads) and mesophyll cells (arrows). (**d** – **f**) stigmas, papillae and subpapillae cells containing polyphenols (arrowheads) and hardly visible nuclei (arrows). (**g** – **i**) ovaries and ovules, high polyphenolic content in the basal part of the ovule and in outer integument cells (arrowheads), arrows – nucellus parenchyma cells, embryo sacs (asterisks); double arrow stands for the extent of cells that were taken into account in the measurement of Alexa488 fluorescence intensity. (**j** – **l**) nectaries, epidermis rich in polyphenols (arrowheads), secretory trichomes (arrows), np nectary parenchyma. Scale bars: b = 10 μm; a, d – f, i, j = 20 μm; c, k, l = 50 μm; g, h = 100 μm

Petals of closed flowers were rich in polyphenols, which were detected in the epidermis and mesophyll cells (Fig. 2a–c). Although phenolic compounds were abundantly present in papillae and subpapillae cells of the stigma, the nuclei were visible (Fig. 2d–f). Within ovaries, the ovules comprised a double-layered integument with tightly adhered nucellus parenchyma cells and embryo sac (Fig. 2g–l). The nectaries were composed of the epidermis, nectary parenchyma, and secretory trichomes in which polyphenolic compounds, were mainly detected (Fig. 2m–o). Similarly to closed flowers, petals of open flowers contained large amounts of phenolic compounds, and the same for stigma papillae and subpapillae cells, where nuclei were “masked” with abundant polyphenol presence (Fig. 3a-f). Part of the nucellus parenchyma cells degenerated, leaving space within the ovule (Fig. 3g–i). The histology of nectaries remained unchanged compared to closed flowers (Fig. 3j–l).

### DNA methylation analysis

**Fig. 4:**
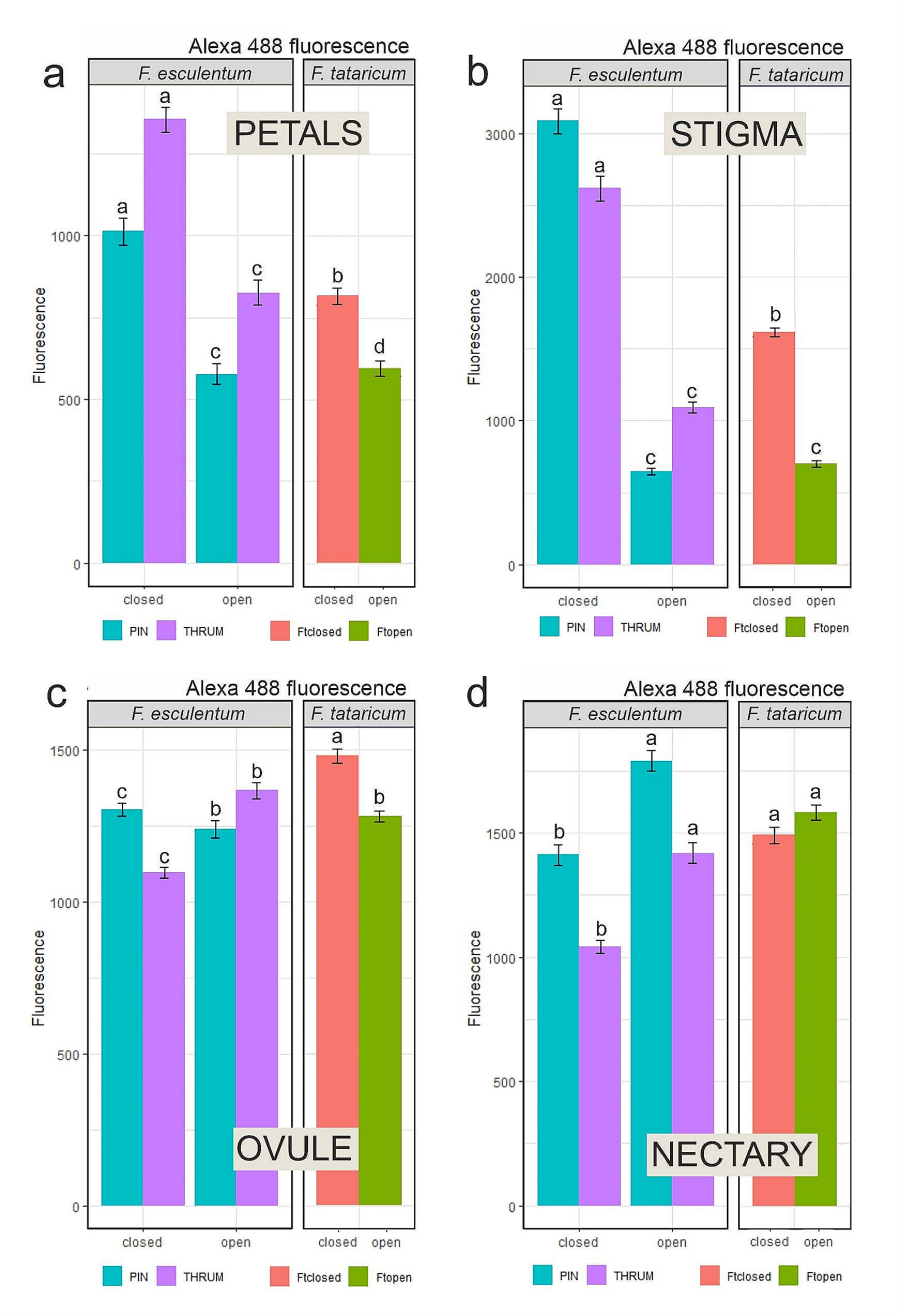
Fluorescence intensity measurements of DNA methylation levels. (**a**) petal; *n* = 500, (**b**) stigma, *n* = 1000, (**c**) ovule, *n* = 1500, and (**d**) nectary, *n* = 1000 parts of the closed and the open flowers of *F. esculentum* and *F. tataricum*. Bars represent standard error; results presented in relative units, letters indicate statistically significant differences between the groups

The highest DNA methylation level in petals from closed flowers was in the Thrum morph; the Pin morph exhibited slightly lower values, whereas the *F. tataricum* open flowers presented the lowest level of this modification. In open flowers, the methylation values decreased in each of the analysed floral parts, but the decrease was the most prominent in Thrum petals (Fig. 4a). Summarising, the *F. esculentum* Thrum morph petals were characterised by the highest starting DNA methylation level and the most eminent decrease; *F. tataricum*, exhibited the lowest DNA methylation value in closed flowers and the lowest decrease during petal development.

Stigma is a pistil component crucial for the first pollination stages. In this floral part, we observed the highest values of the DNA methylation levels in closed flowers among analysed specimens and each flower part and the most considerable reduction in the open flower stage. The highest output DNA methylation level in closed flowers, as well as the decrease (approximately five-fold drop), was noticed in Pin morph (Fig. 4b). Thrum morph was characterised by a lower DNA methylation level concurrently with the lower decrease of the methylation values in comparison to Pin morph (approximately two-fold drop; Fig. 4b). *F. tataricum* stigmas exhibited the lowest starting DNA methylation level among each variant at the closed flower stage, and around two-fold drop in an open flower, which is similar to value decrease observed in Thrum morph (Fig. 4b).

Among the analysed ovules, the DNA methylation level was highest in closed flowers of *F. tataricum*, then in the Pin morph and the lowest in the Thrum morph (Fig. 4c). During flower development, a slight reduction of DNA methylation level was observed for both Pin and Tartary (Fig. 4c) on the contrary to Thrum morph ovules, in which the values of methylation level increased in open flowers (Fig. 4c).

The highest DNA methylation level in nectaries from closed flowers was observed in Tartary buckwheat, lower in Pin, and the lowest in the Thrum morph (Fig. 4d). In contrast to other flower parts (except for the Thrum morph ovule), the nectaries from open flowers had higher values of DNA methylation (compared to values of closed flowers) in all three specimens (Fig. 4d). However, the increase in DNA methylation level was minor, not as prominent as in Pin or Thrum morph. In the opened flowers stage, the *F. esculentum* Pin morph exhibited the highest DNA methylation level, following Tartary, and the lowest values were noted in the Thrum morph.

In conclusion, *F. esculentum* Pin and Thrum morphs differ significantly in DNA methylation levels in the analysed parts of the flower. In the ovule, stigma, and petals of open flowers, the Thrum morph exhibited higher values than the Pin. Results from Tartary buckwheat show some similarities to either Pin or Thrum, depending on the analysed part and stadium of the flower development.

The graphs show global DNA methylation analysis, but within parts such as ovules and nectaries, the DNA methylation level was not uniform in all nuclei. For example, in ovules, the nuclei from the embryo sac showed very low or no DNA methylation in contrast to other ovule nuclei (Additional File 7).

### Gene expression analysis

**Fig. 5:**
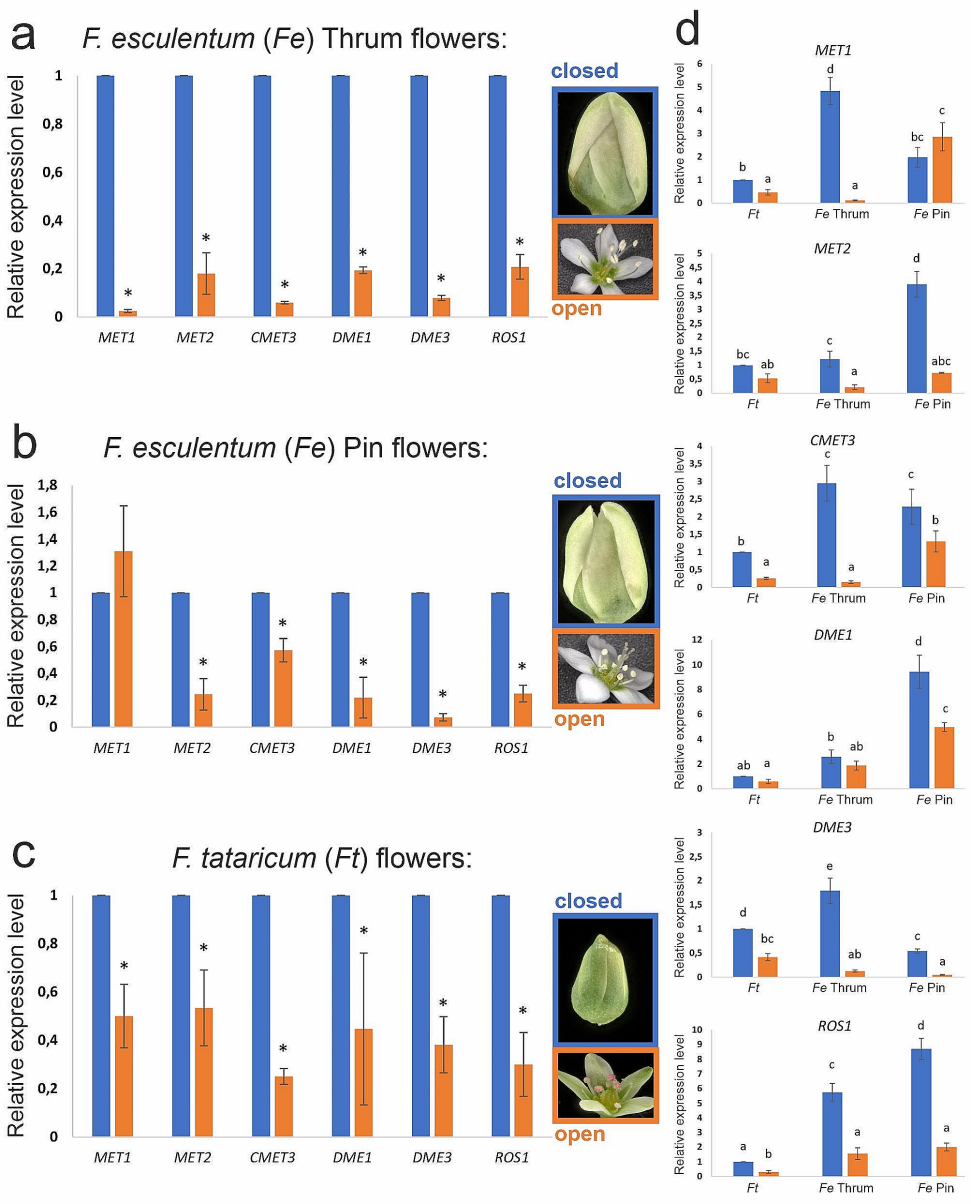
Expression level of *MET1*, *MET2*, *CMET3*, *DME1*, *DME3*, and *ROS1* in closed and open flowers, (**a**, **d**) Thrum and (**b**, **d**) Pin of *F. esculentum* and (**c**, **d**) *F. tataricum*. The expression level of genes in open flowers was calibrated to expression in closed flowers of the same type and species (a, b, c). * - values significantly different from closed flowers of the same type and species (a, b,c) (*p* < 0.05; *n* = 3; means ± SD are given). The expression level of genes in all types and species was calibrated to expression in closed flowers of the *F. tataricum* (d). Different letters indicate a significant difference between flower type and species according to Tukey’s HSD test (*p* < 0.05; *n* = 3; means ± SD are given)

We analysed the expression level of genes related to DNA methylation (*MET1, MET2, CMET3*) and demethylation (*DME1, DME3, ROS1*) in closed and open Pin and Thrum flowers of *F. esculentum* and flowers of *F. tataricum*. The results showed the down-regulation of all genes in Thrum flowers (Fig. 5a). The most intense decrease in expression was observed for *MET1*, which was over 45 times lower in the open than in closed Thrum flowers. In contrast to Thrum flowers, the expression of *MET1* in open Pin flowers was the same as in closed ones (Fig. 5b). The decreased transcript level of the rest of the analysed genes was observed in open Pin flowers. The highest reduction was characteristic for the *DME3* gene (14 times lower in open than in closed Pin flowers). The decreased transcript level of almost all analysed genes was observed in open flowers of *F. tataricum* (Fig. 5c). In general, during the development of flowers, the expression of genes encoding DNA methylases and demethylases was decreased.

In addition, we analysed the expression of the genes between flower types and species (Fig. 5d). The expression of all genes is higher (from 2.5 to over 9 times higher) in closed flowers of *F. esculentum* than in *F. tataricum*. Moreover, we observed the difference in expression of genes between Pin and Thrum flowers, both closed and open. *MET2*, *DME1*, and *ROS1* expression were lower in Pin than in Thrum flowers. The other three analysed genes (*MET1, CMET3, DME3*) are characterised by higher transcript levels in Thrum than in Pin flowers.

## Discussion

The objective of this study was to compare the DNA methylation status between the Pin and Thrum flower components of *F. esculentum*, with that of self-pollinating species like *F. tataricum* in order to shed light on how this epigenetic mark may have a role in the floral development of the *Fagopyrum* species. *F. tataricum* flowers are smaller than those of *F. esculentum* (Pin, Thrum). In *F. esculentum* open flowers, Pin flowers feature a long style and shorter anther stamens, while Thrum has a short style and longer anther stamens. Open flowers of *F. tataricum* were characterised by green petals, style, and anthers of similar length. The heteromorphic heterostyly of *F. esculentum* is a significant limitation in breeding [15, 64]. Around a few thousand phenolic structures in the plant kingdom were reported – ranging from single, aromatic-ringed compounds to complex structures [65]. In *F. esculentum*, a total of 60 different phenolic substances were identified, with the highest number found in the flower part of the plant [66]. In tea, another species rich in this type of secondary metabolites, the accumulation of phenolics in leaves was developmentally regulated during bud or first leaf expansion [67]. Moreover, in tobacco, the subsequent stages of flower development were correlated with tissue contents of polyphenols and activities of L-phenylalanine ammonia-lyase, polyphenoloxidase and peroxidase [68]. Also, a large amount of polyphenols was accumulated in tobacco pistil [68]– both reports are consistent with our observations. Although the occurrence of phenolic compounds was not the main objective of our study, it cannot be excluded that DNA methylation changes also affect polyphenolic accumulation, as was reported for *Salvia* sp [69].

DNA methylation, one of the most researched epigenetic modifications, has been linked with regulating chromatin structure and gene expression [16, 25]. It is involved in various developmental processes in flowers, including developing the floral organs, regulating the flowering time, and floral patterning [19]. DNA methylation impacts floral development by modulating the expression of flowering genes via epigenetic alterations. Promoter methylation typically correlates with the suppression of gene expression, while the effects of gene body methylation remain ambiguous, with studies reporting both positive and negative associations [20, 70, 71]. A conducted study reported that DNA methylation in non-promoter, intergenic regions and gene bodies facilitates gene expression. Non-promoter DNA methylation appears crucial for preserving the active chromatin states of genes [26, 72, 73]. It has been previously demonstrated that this epigenetic mark is essential in flower development and that its enrichment fluctuates in various developmental stages [28, 35, 41]. Here, we demonstrated the differences in the level of DNA methylation in flowers at various developmental stages in two buckwheat species, *F. tataricum* with homostylous flower type and *F. esculentum* with heterostylous flowers. Only the nectaries from open flowers had higher values of DNA methylation in comparison to results from closed flowers. DNA methylation values decreased in petals, stigmas and ovules (except for Thrum morph) of each analysed variant in open, developed flowers. In studies conducted on lotus (*Nelumbo nucifera*), the stamen petaloid exhibited a global decrease in DNA methylation levels [74]. Studies comparing DNA methylation patterns in ovules of the female-sterile rice and the wild-type, displayed a slightly lower whole-genome methylation level [75]. Research on hazel ovaries reported the reduction in DNA methylation levels of the ovule after pollination, indicating that the epigenetic mark is a crucial player in post-pollination stages [76]. It is known that stigma and pollen molecular cross-talk are crucial for successful pollination [77, 78]. During flower maturation, the papillae and sub-papillae cells of the stigma undergo histo- and biochemical changes to prepare for pollen reception, which involves cell loosening or exudate synthesis [79]. In our research, we observed a significant decrease in DNA methylation levels between stigmas of closed and open *F. esculentum* and *F. tataricum* flower; however, the divergence between DNA methylation levels in Pin and Thrum was the most prominent. This trait may point to differences between homo- and distylous species.

Studies conducted on *Lilium longiflorum* cv. Gelria focused on the analysis of the DNA methylation in pollen and reported that the vegetative nucleus in mature pollen grains was heavily methylated and that dramatic nonreplicative demethylation occurred during the pollen tube’s development. In conclusion, it was established that DNA hypomethylation ensures the survival of pollen grains without external sources of nutrients until they reach the stigma [80]. Since open flowers in *Fagopyrum* already have produced pollen grains, this might have contributed to the global reduction in DNA methylation levels. The reduction in DNA methylation is correlated with the down-regulation of genes coding DNA methyltransferases. In the Thrum open flower, the expression of *MET1* was over 45 times lower in the open than in closed Thrum flowers.

In contrast to Thrum flowers, the expression of *MET1* in open Pin flowers was the same as in closed ones. It seems that in Pin open flowers, the decrease in DNA methylation level might be related to the down-regulation of genes other than *MET1* methyltransferase, such as *MET2* or *CMET3*. The differential expression of methyltransferases is observed between various tissues [81]; for example, in rice, the transcript of *MET1b* accumulates more abundantly than those of *MET1a* in many different tissues [82]. In our research, the decreased transcript level of the rest of the analysed genes was observed in open Pin flowers. *ROS1* encodes a nuclear protein containing an endonuclease III domain, exhibiting bifunctional DNA glycosylase/lyase activity specifically targeting methylated DNA while leaving unmethylated DNA unaffected [27]. It might explain why the levels of *ROS1* expression are reduced in open *Fagopyrum* flowers, which exhibit reduced DNA methylation levels. Lower transcript accumulation of genes engaged in DNA demethylation was observed in open flowers of all analysed flower types and species. The DNA methylation level of analysed tissue is an interplay between DNA methylation and active demethylation. Methylation strongly affects flowering-related genes’ expression and mobility of transposons [83]. Effective demethylation occurs on the maternal central cell before fertilisation, and in the endosperm, maternal alleles are less methylated than paternal ones [84]. Among others, imprinted genes such as *FLOWERING WAGENINGEN* (*FWA*), *MEDEA* (*MEA*), and *FERTILIZATION-INDEPENDENT SEED 2* (*FIS2*) are methylated and silenced in the spermatic cell, so after fertilisation, only maternal alleles are expressed in the endosperm [85]. Thus, the methylation and demethylation of specific DNA sequences are essential in regulating genes for the proper development of flowers and embryos.

Acquisition of the flowering abilities and subsequent development is precisely governed by the synchronised and specific expression patterns of microRNAs associated with flowering in plants [86, 87]. MicroRNAs represent a new category of intrinsic molecules that modulate gene expression, particularly during the pathways of flower development in plants [83, 88, 89]. Research has shown that the relative expression levels of several potential miRNAs remain notably consistent while governing the flowering time phenotype in a spatial and temporal context [90].

## Conclusions

The current study illustrates the distinct DNA methylation patterns observed between the Pin and Thrum flower components of *F. esculentum*, as compared to self-pollinating species like *F. tataricum*, offering insights into the potential role of this epigenetic modification in the floral development of *Fagopyrum* species. Decreased overall DNA methylation and expression of genes connected with that epigenetic mark in open, developed flowers of both species might suggest that the demethylation is required to activate the expression of genes involved in floral development. Understanding the role of DNA methylation in flowers is crucial for unravelling the molecular mechanisms underlying floral development, adaptation to environmental changes, and the regulation of floral traits. It provides insights into how epigenetic modifications contribute to the diversity and complexity of flower morphology and function. Comparing the global methylation of DNA and expression of genes in both morphs of *F. esculentum*, as well as with a closely related, homostylous species, *F. tataricum* can lead to deciphering and understanding the differences between them and influence the self-incompatibility phenomenon to create self-compatible varieties. Our analyses offer insights into the potential roles of DNA methylation in gene expression and serve as valuable resources for further exploration of the genetic pathways governing flower development.

### Electronic supplementary material

Below is the link to the electronic supplementary material.

**Additional File 1**: Graphical depiction of the experimental design

**Additional File 2**: Excel table containing raw numerical data for DNA methylation

**Additional File 3**: R-Studio script for statistical analysis and graphical depiction of the results

**Additional File 4**: Excel table containing forward and reverse primers for the RT-qPCR analysis

**Additional File 5**: BioRender certificate confirming the publication rights for Additional File

**Additional File 6**: Histology of anthers from closed (a – c) and open (d – f) flowers (not included in DNA methylation analysis)

**Additional File 7**: Selected images captured with the Olympus FV1000 confocal system

## Data Availability

All data generated or analysed during this study are included in this published article (and its additional information files).

## Abbreviations

BSA: Bovine albumin serum
CMT3: *CHROMOMETHYLASE 3*
DAPI: 4’,6-diamidyno-2-fenyloindol
*DME*: *Demeter*, *5-methylcytosine glycosylase*
*DML2*: *DME-like 2*, *5-methylcytosine glycosylase*
*DML3*: *DME-like 3*, *5-methylcytosine glycosylase*
*DRMs*: *DOMAINS REARRANGED METHYLTRANSFERASEs*
*MET1*: *DNA METHYLTRANSFERASE 1*
PBS: 1x phosphate-buffered saline
*ROS1*: *Repressor of silencing 1*, *5-methylcytosine glycosylase*
*S-ELF3*: *S-LOCUS EARLY FLOWERING 3*
TBO: Toluidine blue O
