# Supplementary figures and images for: DNA methylation analysis of floral parts revealed dynamic changes during the development of homostylous Fagopyrum tataricum and heterostylous F. esculentum flowers
Source: BMC Plant Biol. 2024 May 23;24:448. doi: 10.1186/s12870-024-05162-w (PMC11112930; doi:10.1186/s12870-024-05162-w)

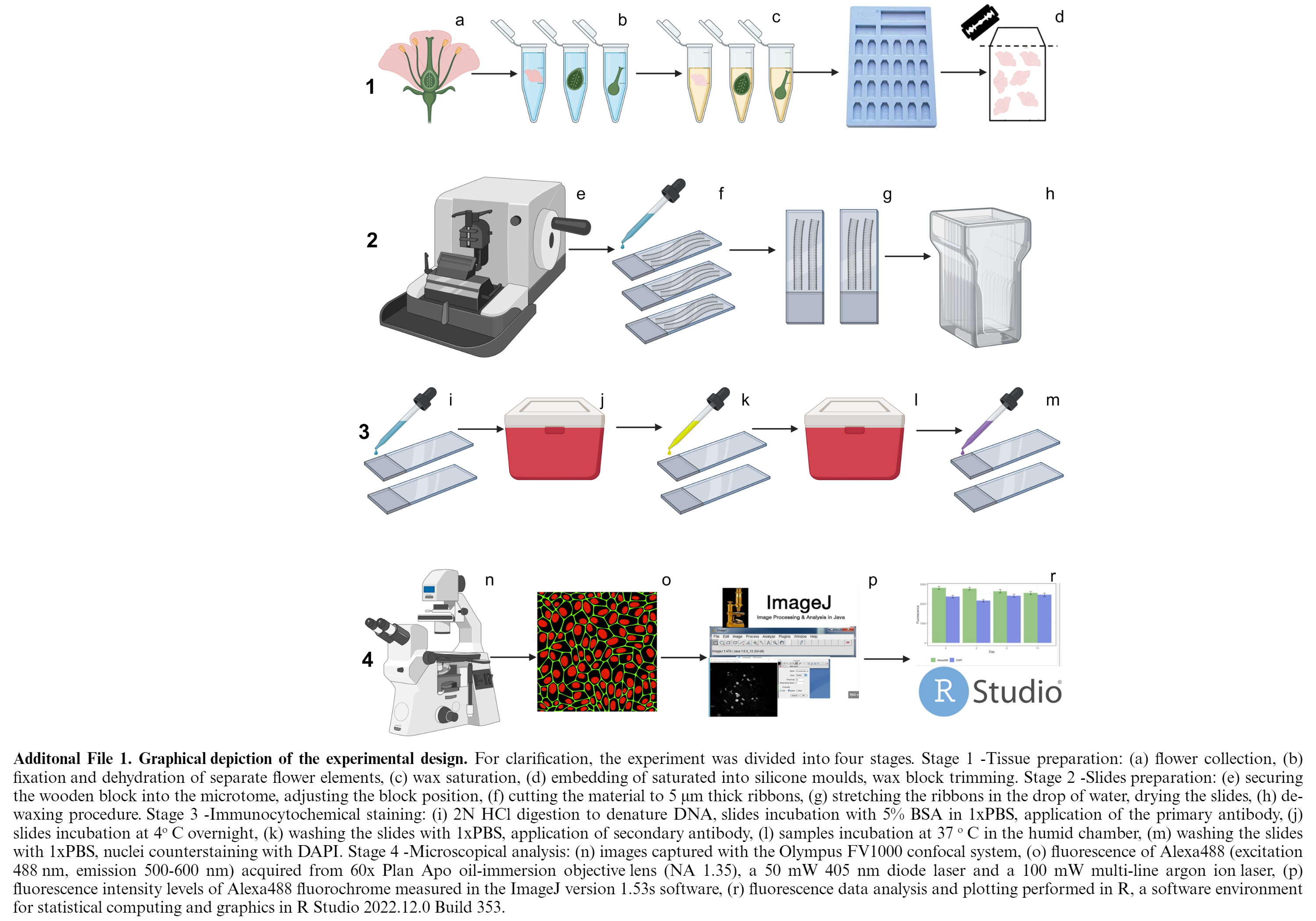

Supplement: Supplementary file 1 — Additional File 1: Graphical depiction of the experimental design [file 12870_2024_5162_MOESM1_ESM.jpeg]

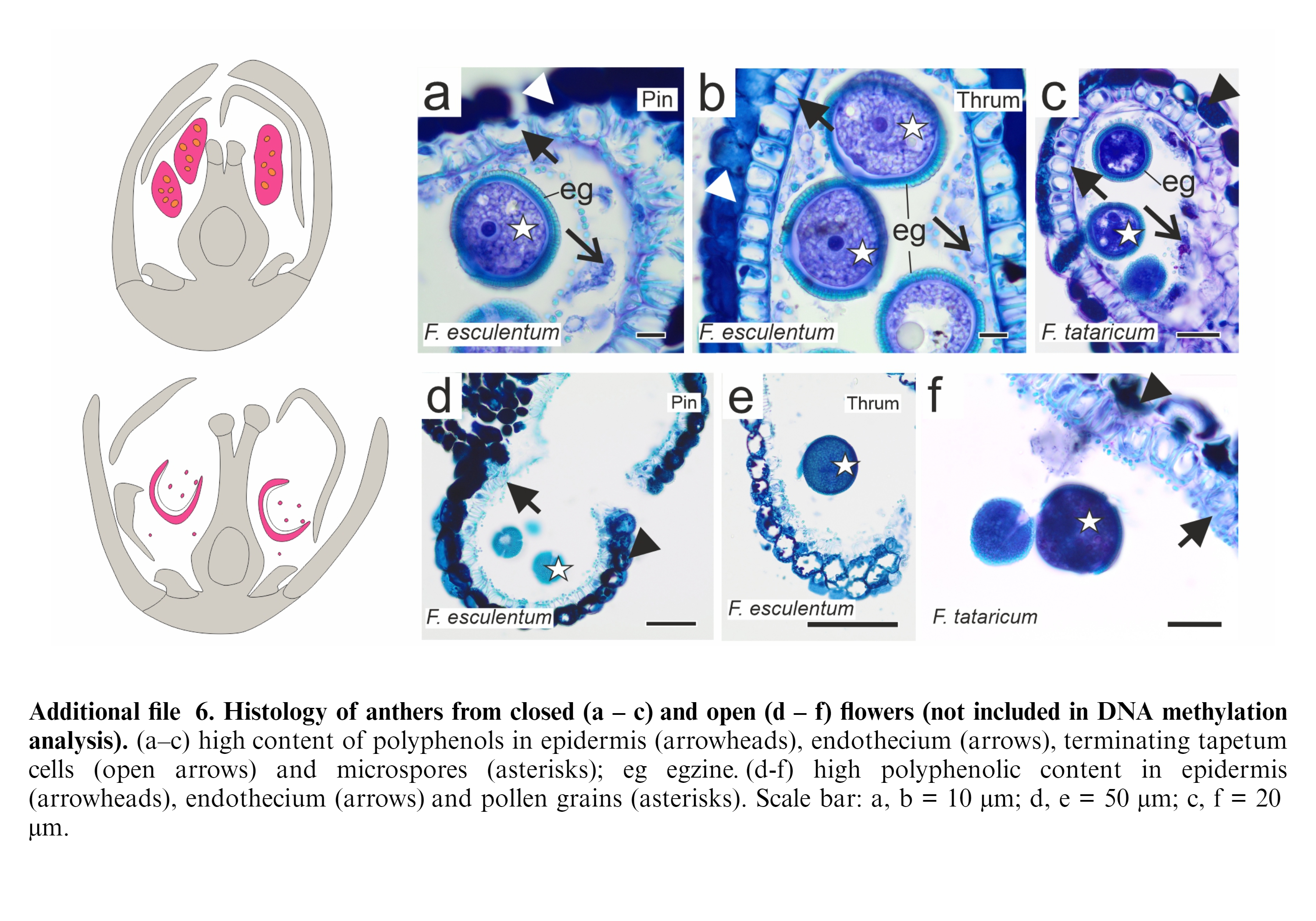

Supplement: Supplementary file 6 — Additional File 6: Histology of anthers from closed (a – c) and open (d – f) flowers (not included in DNA methylation analysis) [file 12870_2024_5162_MOESM6_ESM.jpeg]

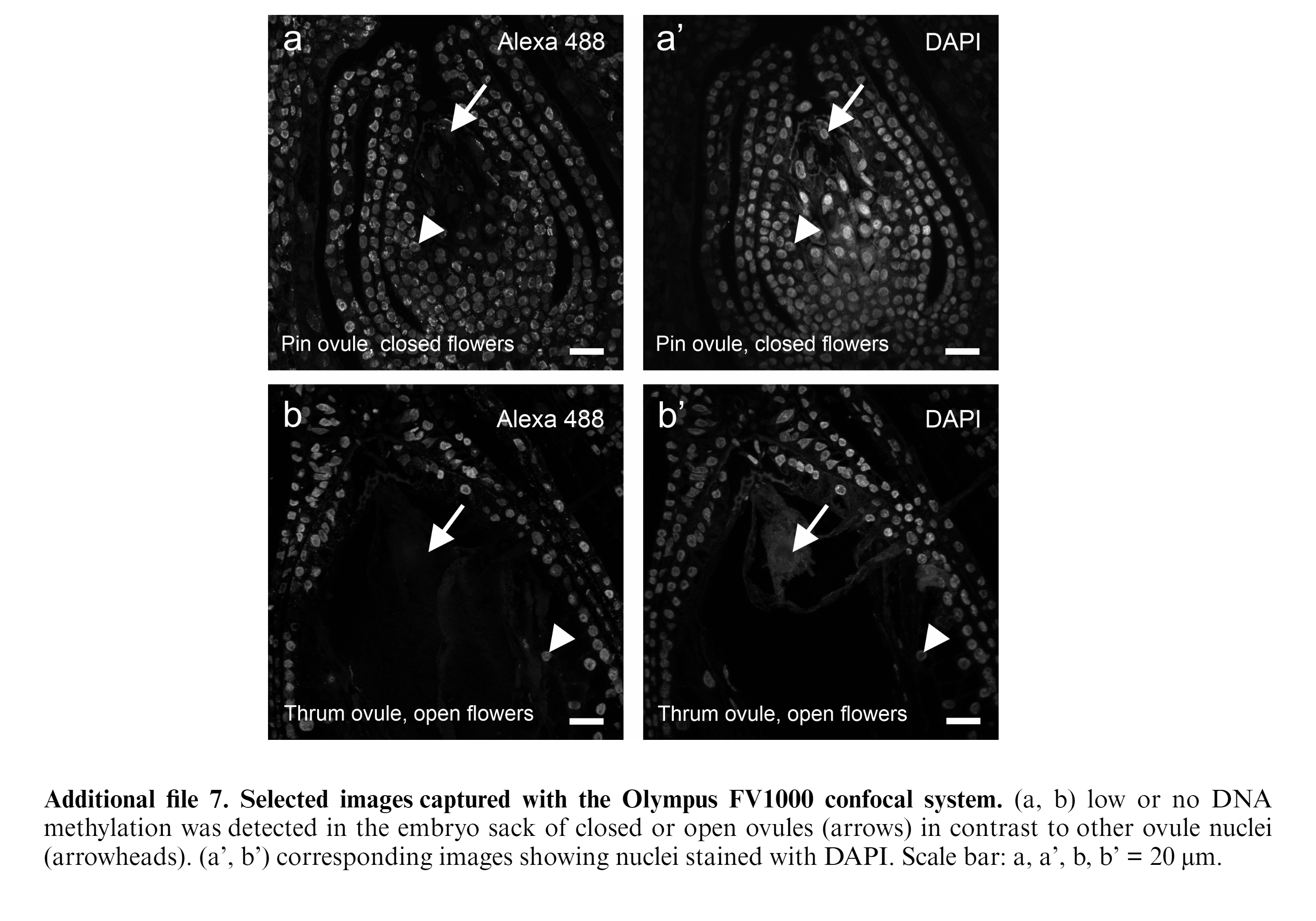

Supplement: Supplementary file 7 — Additional File 7: Selected images captured with the Olympus FV1000 confocal system [file 12870_2024_5162_MOESM7_ESM.jpeg]
