# Supplementary material for: DNA methylation analysis of floral parts revealed dynamic changes during the development of homostylous Fagopyrum tataricum and heterostylous F. esculentum flowers
Source: BMC Plant Biol. 2024 May 23;24:448. doi: 10.1186/s12870-024-05162-w (PMC11112930; doi:10.1186/s12870-024-05162-w)
Supplement: Supplementary file 3 — Additional File 3: R-Studio script for statistical analysis and graphical depiction of the results [file 12870_2024_5162_MOESM3_ESM.docx]

**Additional file 3.** R-Studio script for statistical analysis and graphical depiction of the results.

**Statistical analysis and visualization with R**

1. Open R Studio, start a new R script in which you will write the required code by clicking on Packages in the task bar in the right bottom and download the required packages: tidyverse, readxl and agricolae
2. Load the libraries for these packages by using “library(packagename)”
3. Load the excel file with the data by using “dataset <- read_excel(“filename.xlsx”, header=T)”. The excel file should contain a column that groups the nuclei, for example the column “nectary”, a column with the fluorescence values for open flowers (name can be “open”) and another column with the fluorescence values for closed flowers (“closed”)
4. Make sure the columns have the correct class by using “as.numeric(dataset$open)” and “as.numeric(dataset$closed)”. The grouping column should be “as.character(dataset$nectary)”
5. To calculate mean fluorescence with standard deviation, execute the following code: “dataset_open <- dataset %>% group_by(nectary) %>%

summarise(mean = mean(na.omit(open)),

sd = sd(na.omit(open)),

state = “open)

dataset_closed <- dataset %>% group_by(nectary) %>%

mean = mean(na.omit(closed)),

sd = sd(na.omit(closed)),

state = “closed”)

dataset_closed_open <- rbind(dataset_open, dataset_closed)”

1. Next, use ANOVA and Tukey’s HSD test to test for significant differences between the groups, in this case between different types of flowers:

“anovaopen <-aov(dataset$open ~dataset$nectary)

summary(anovaopen)

tukeyopen <- HSD.test(anovaopen, trt="dataset$nectary")

tukeyopen”

1. The output shows each group (nectary) with a letter. There is a significant difference (p<0.05) between groups, when they have different letters (e.g. one group is “a” and the other is “b”).
2. Repeat steps 6 and 7 and replace “open” in the code with “closed” to get the test results for fluorescence of closed flowers
3. Save the significance levels in a dataset like in the following example:

“significances <- data.frame(nectary = c("PIN", "PIN", "THRUM", "THRUM"),

state = c("open", "closed", "open", "closed"),

significances = c("a", "a", "a", "b"))”

1. Merge this with the statistics dataset:

“final_data <- merge(dataset_closed_open, significances)”

1. Reorder the data to display the x axis in the order you want to have it in the future plot:

“final_data$nectary <- ordered(final_data$nectary , levels=c("PIN", "THRUM"))”

1. To plot the fluorescence of open flowers as bar plots with error bars, use the following code:

“ggplot(final_data, aes(x=factor(nectary), y=mean))+

geom_col(position = "dodge", aes(fill=state))+

geom_errorbar(mapping=aes(ymin=mean - sd, ymax=mean + sd), show.legend = F, position = position_dodge2(padding=0.8))+

labs(x="Nectary", y="Fluorescence", title="Alexa488 fluorescence")”

1. Executing this block of code will display the statistical analysis results and the plot in the “Plots” panel of R Studio where you can click on “Export” in order to save the plot as a file or to the clipboard
