# Supplementary material for: DNA methylation analysis of floral parts revealed dynamic changes during the development of homostylous Fagopyrum tataricum and heterostylous F. esculentum flowers
Source: BMC Plant Biol. 2024 May 23;24:448. doi: 10.1186/s12870-024-05162-w (PMC11112930; doi:10.1186/s12870-024-05162-w)
Supplement: Supplementary file 5 — Additional File 5: BioRender certificate confirming the publication rights for Additional File [file 12870_2024_5162_MOESM5_ESM.pdf]

49 Spadina Ave. Suite 200
Toronto ON M5V 2J1 Canada
www.biorender.com

Confirmation of Publication and Licensing Rights

May 9th, 2024
Science Suite Inc.

Subscription:	*Institution*
Agreement number:	*FI26SPHNPY*
Journal name:	*BMC Plant Biology*

To whom this may concern,

This document is to confirm that Alexander Betekhtin has been granted a license to use the BioRender content, including icons, templates and other original artwork, appearing in the attached completed graphic pursuant to BioRender's Academic License Terms. This license permits BioRender content to be sublicensed for use in journal publications.

All rights and ownership of BioRender content are reserved by BioRender. All completed graphics must be accompanied by the following citation: “Created with BioRender.com”.

BioRender content included in the completed graphic is not licensed for any commercial uses beyond publication in a journal. For any commercial use of this figure, users may, if allowed, recreate it in BioRender under an Industry BioRender Plan.

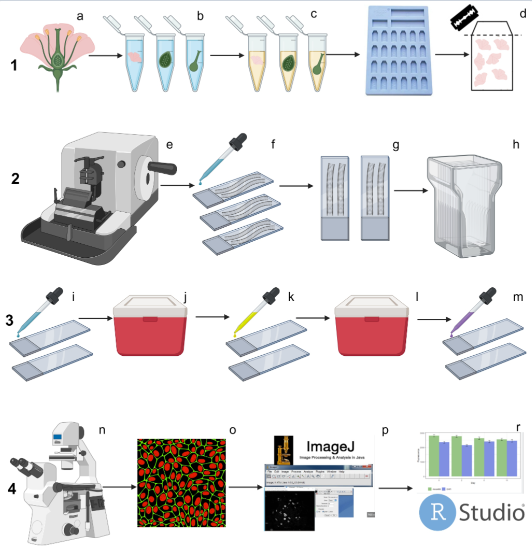

Additonal File 1. Graphical depiction of the experimental design. For clarification, the experiment was divided into four stages. Stage 1 -Tissue preparation: (a) flower collection, (b) fixation and dehydration of separate flower elements, (c) wax saturation, (d) embedding of saturated into silicone moulds, wax block trimming. Stage 2 -Slides preparation: (e) securing the wooden block into the microtome, adjusting the block position, (f) cutting the material to 5 μm thick ribbons, (g) stretching the ribbons in the drop of water, drying the slides, (h) de-waxing procedure. Stage 3 -Immunocytochemical staining: (i) 2N HCl digestion to denature DNA, slides incubation with 5% BSA in 1xPBS, application of the primary antibody, (j) slides incubation at 4° C overnight, (k) washing the slides with 1xPBS, application of secondary antibody, (l) samples incubation at 37 ° C in the humid chamber, (m) washing the slides with 1xPBS, nuclei counterstaining with DAPI. Stage 4 -Microscopical analysis: (n) images captured with the Olympus FV1000 confocal system, (o) fluorescence of Alexa488 (excitation 488 nm, emission 500-600 nm) acquired from 60x Plan Apo oil-immersion objective lens (NA 1.35), a 50 mW 405 nm diode laser and a 100 mW multi-line argon ion laser, (p) fluorescence intensity levels of Alexa488 fluorochrome measured in the ImageJ version 1.53s software, (r) fluorescence data analysis and plotting performed in R, a software environment for statistical computing and graphics in R Studio 2022.12.0 Build 353.

For any questions regarding this document, or other questions about publishing with BioRender refer to our BioRender Publication Guide, or contact BioRender Support at support@biorender.com.
